# Inside the April 2026 Issue

**DOI:** 10.24908/pocusj.v11i01.20878

**Published:** 2026-04-22

**Authors:** Benjamin T. Galen

**Affiliations:** Professor of Medicine, Albert Einstein College of Medicine and Montefiore Medical Center, Bronx, NY, USA, Editor-in-Chief, POCUS Journal

**Keywords:** April 2026, Commentary

Dear Readers,

In clinical medicine, we course correct constantly: re-evaluating a diagnosis, revising a management plan, acknowledging that new data no longer support our original conclusions. It is part of the work. In academic medical publishing, these same practices must also apply. This month, POCUS Journal publishes its first Corrigendum: *Normal Anatomy Mimicking an Abdominal Aortic Dissection*, Kee et al. on page 50. There is a particular kind of humility in publishing a correction. Not because something went terribly wrong, but because something went right: the system worked and the community came together. The conclusions in a case report from last year were re-evaluated by Dr. Tanping Wong of our editorial board and the authors graciously accepted the feedback. The POCUS community has a lot of integrity and everyone who helped make this corrigendum possible represents our unwavering commitment to accurate image interpretation; a fascinating mimic of abdominal aortic dissection was attributed to artifact but later concluded to be due to the way that the crus of the diaphragm appears in today's high-resolution scans.

Publishing this corrigendum reflects POCUS Journal's value for prioritizing transparency and we hope that the corrected case report published in the April issue strengthens—not weakens—the trust our readers place in the POCUS journal. We are grateful that the authors eagerly accepted the opportunity to correct their case report, which will enable anyone who reads it to properly understand how normal anatomy can create a mimic of abdominal aortic dissection on POCUS examination.

Beyond this milestone, the April issue reflects the continued maturation of POCUS as both a clinical tool and an academic discipline. The contributions in this issue move comfortably between bedside application and broader systems thinking. In addition to riveting cases with high-quality images, the April issue contains exciting new applications for POCUS as well as novel POCUS curricula. Please Enjoy.

Sincerely,

Benjamin T. Galen, MD

Professor of Medicine

Albert Einstein College of Medicine and Montefiore Medical Center, Bronx, NY, USA

Editor-in-Chief,

POCUS Journal

**Figure:**
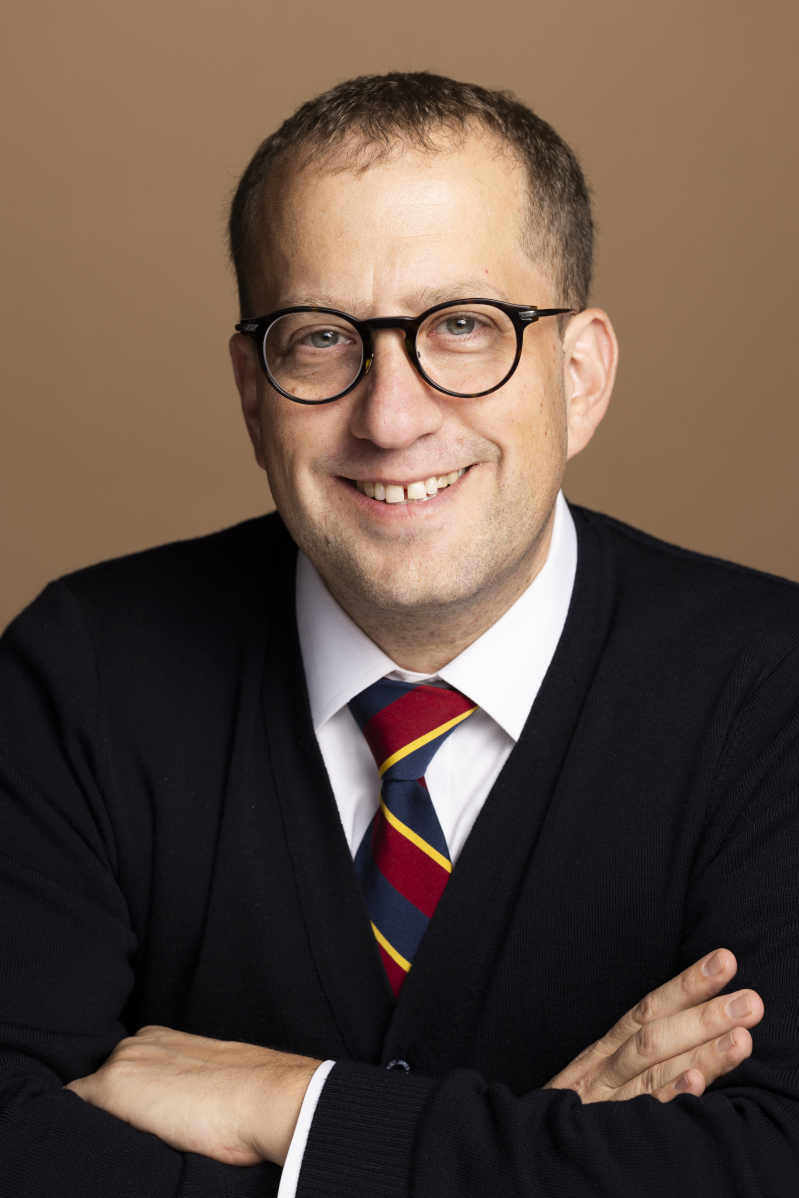
Benjamin T. Galen, Editor in Chief, POCUS Journal

